# Mutational Landscape of Bone Marrow CD19 and CD138 Cells in Waldenström Macroglobulinemia (WM) and IgM Monoclonal Gammopathy of Undetermined Significance (IgM MGUS)

**DOI:** 10.1002/cam4.70525

**Published:** 2024-12-23

**Authors:** Alessandra Trojani, Alessandro Beghini, Luca Emanuele Bossi, Marta Rachele Stefanucci, Cassandra Palumbo, Antonino Greco, Annamaria Frustaci, Barbara Di Camillo, Roberto Cairoli

**Affiliations:** ^1^ Niguarda Hospital Department of Hematology and Oncology Milano Italy; ^2^ Department of Health Sciences University of Milano Milano Italy; ^3^ A.R.N.A.S. Ospedali Civico Di Cristina Benfratelli Palermo Italy; ^4^ Department of Information Engineering University of Padova Padova Italy

**Keywords:** IgM monoclonal Gammopathy of undetermined significance, mutations, NGS, Waldenström Macroglobulinemia

## Abstract

**Background:**

Despite recurrent and activating mutations, including *MYD88*, *CXCR4*, *ARID1A*, *KMT2D*, and *CD79B* were identified, the genetic basis for Waldenström's Macroglobulinemia (WM) and the risk of progression of IgM MGUS to WM remain to be fully elucidated.

**Methods:**

We investigated the mutation status of WM (*n* = 8), sWM (*n* = 7), and IgM MGUS (*n* = 5) patients, by performing high‐throughput targeted AmpliSeq NGS on 117 target genes. Specifically, we analyzed the CD19+ cells from 15 WM/sWM patients and five IgM MGUS patients. We also analyzed the CD138+ cells from four WM/sWM patients and two IgM MGUS patients.

**Results:**

We detected the classic mutation *MYD88*L265P in 93% of WM/sWM and in 60% of IgM MGUS patients. The *CXCR4*S338Ter mutation was identified in 26% of WM/sWM patients, whereas it was undetectable in IgM MGUS subjects. Interestingly, we identified new mutated genes, including *WNK2* somatic mutations affecting 46% of WM/sWM patients, for which a recurrent allelic variant (V1635Ter) was observed in this cohort. Moreover, sequencing evaluation revealed recurrently frameshift or missense mutations involving *NFKB2* (L473Afs) in 60% of IgM MGUS and 20% of WM/sWM, *PTPN13* (P1546Tfs) in 20% of IgM MGUS and 7% of WM/sWM, *CARD11* (S622del) in 20% of IgM MGUS and 20% of WM/sWM, *KMT2C* (I823T) in all IgM MGUS and 93% of WM/sWM, and *ATM* in 20% of IgM MGUS and 47% of WM/sWM patients.

**Conclusion:**

In conclusion, we uncovered new insights into the mutational landscape of WM, depicting a more complex involvement of the NF‐kB pathway, and providing evidence of the recurrence of some variants (*MYD88*, *IL17RB*, *NFKB2*, *ATM*, *CARD11*, *PTPN13*, and *WNK2*) also in IgM MGUS.

## Graphical abstract

NGS analysis of selected genes in CD19+ and CD138+ cells of WM and IgM MGUS patients.

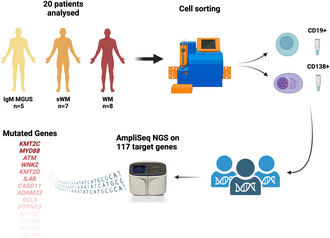

## 1 Introduction

Waldenström's Macroglobulinemia (WM) is an incurable B‐cell neoplasm characterized by serum monoclonal immunoglobulin M (IgM) and clonal lymphoplasmacytic cells infiltrating the bone marrow (BM). Smoldering WM (sWM) is the asymptomatic/indolent form with a high risk of progressing to symptomatic WM requiring treatment, whereas IgM monoclonal gammopathy of undetermined significance (IgM MGUS) is an early precursor stage of WM. IgM MGUS is an asymptomatic form with an overall risk of progression of 1.5%–2% per year and approximately 18% at 10 years to sWM or other lymphoproliferative disorders [1].

The molecular landscape of WM is primarily characterized by recurrent mutations in *MYD88* (MYD88 Innate Immune Signal Transduction Adaptor) and *CXCR4* (C‐X‐C Motif Chemokine Receptor 4) genes. *MYD88* and *CXCR4* mutations play a crucial role in the diagnosis and prognostic stratification of WM patients and have therapeutic implications [1]. *MYD88* L265P mutation prevalence varies from 54% to 87% in IgM MGUS [2, 3].

*MYD88* somatic mutations trigger NF‐κB (nuclear factor kappa‐light‐chain‐enhancer of activated B cells) signaling pathway through BTK (Bruton Tyrosine Kinase) and IRAK1/IRAK4 (Interleukin 1 Receptor Associated Kinase 1/Interleukin 1 Receptor‐Associated Kinase 4), leading to the activation of B cell proliferation [1, 4].

*CXCR4* mutations are mainly considered a later subclonal event in a smaller number of WM patients (30%–43%) and IgM MGUS patients (17%–35%), respectively [5, 6]. These mutations primarily activate *RAS*, *Akt*, and NF‐κB signaling pathways, mediating B cell and plasma cell homing to the bone marrow [7]. IgM MGUS patients exhibit a significantly lower number of mutations than WM patients, suggesting that multiple genetic events are required for progression into WM or other lymphoproliferative disorders [8]. In addition to genomic alterations, changes in the gene expression profiles of B cells and plasma cells in WM and IgM MGUS have been investigated by various research studies [9].

Recent advances in exploring the mutational profile of WM revealed additional genetic aberrations in some other genes. Among them, missense, nonsense, and frameshift mutations in *ATM* (ATM Serine/Threonine Kinase), *CARD11* (Caspase Recruitment Domain Family member 11), *CD79B* (CD79b Molecule), *NFKB2* (Nuclear Factor Kappa B Subunit 2), *PTPN13* (Protein Tyrosine Phosphatase Nonreceptor Type 13), *KMT2C* (Lysine Methyltransferase 2C), *KMT2D* (Lysine Methyltransferase 2D), and *ARID1A* (AT‐Rich Interaction Domain 1A) were described in WM patients [1, 2, 10, 11, 12].

Previous studies have focused on understanding transcriptomic alterations by analyzing bone marrow clonal B cells and plasma cells in comparison to their normal cell counterparts. Gene expression changes could modulate the progression from IgM MGUS to WM [13, 14, 15]. Our previous research has identified a small transcriptome gene set signature demonstrating nine genes including *HIST1H1B* (H1.5 Linker Histone, Cluster Member), *EZH2* (Enhancer Of Zeste 2 Polycomb Repressive Complex 2 Subunit), *CHEK1* (Checkpoint Kinase 1), *LEF1* (Lymphoid Enhancer Binding Factor 1), *ADAM23* (ADAM Metallopeptidase Domain 23), *RASGRP3* (RAS Guanyl Releasing Protein 3), *ADRB2* (Adrenoceptor Beta 2), *PIK3AP1* (Phosphoinositide‐3‐Kinase Adaptor Protein 1), and *CDHR3* (Cadherin Related Family Member 3), which showed similar expression levels between WM and IgM MGUS compared to healthy subjects (CTRLs). This could suggest a potential molecular signature that could predict the risk of progression from IgM MGUS to WM [16].

Despite the fact that several studies identified recurring mutations in various genes and gene expression differences between WM and IgM MGUS patients, further investigations are needed to comprehensively understand the genomic and transcriptional landscape involved. Furthermore, the impact of the mutational status in the early stages of the disease and the underlying biological mechanisms responsible for the progression from the indolent to the symptomatic form are not fully understood at this time.

In this study, we conducted next‐generation sequencing (NGS) sequencing of 117 target genes selected from those already known to be mutated in WM and IgM MGUS, as well as from other genes that have not been previously screened but suggested in our previous transcriptome analyses on BM CD19+ and CD138+ cells of patients with WM and IgM MGUS.

## 2 Methods

### 2.1 Patients

We collected BM samples from 20 patients as follows: WM (*n* = 8), sWM (*n* = 7), and IgM MGUS (*n* = 5) patients, respectively. The clinical characteristics of these patients are presented in Table 1. We isolated mononuclear cells (MNCs) from the BM samples (volume ranging from 6 to 10 mL) using Ficoll density gradient centrifugation at 800 rpm for 20 min. Subsequently, BM CD19+ cells and CD138+ cells were isolated from the BM MNCs using Human CD19 MicroBeads and Human CD138 MicroBeads according to the manufacturer's instructions (Miltenyi Biotec, Milan, Italy) [16]. The selected BM CD19+ and BM CD138+ cells were resuspended in 50 μL of RNAlater (Thermo Fisher Scientific, Milan, Italy) and stored at −80°C. DNA was extracted from both cell types using MagMax‐96 for Microarrays (Thermo Fisher Scientific) following the manufacturer's instructions. The quantification of DNAs was assessed on Qubit 4 fluorometer in combination with Qubit dsDNA HS Assay Kit (Thermo Fisher Scientific, #Q32854).

**TABLE 1:** Clinical characteristics of WM and IgM MGUS patients.

Variable	Total (*n* = 20)	WM (*n* = 15)	IgM MGUS (*n* = 5)
Median age at diagnosis in years (range)	67 (46–79)	66 (46–79)	67 (65–74)
Sex, *n* (%)			
Male	10 (50)	7 (47)	3 (60)
Female	10 (50)	8 (53)	2 (40)
Median % of bone marrow involvement (range)	22.5 (0–90)	30 (0–90)	2 (0–5)
M‐protein level (g/dl) median (range)	1.15 (0.30–7.00)	1.20 (0.40–7.00)	0.40 (0.30–1.20)
Light chain			
k, *n* (%)	12 (60)	8 (53)	4 (80)
l, *n* (%)	6 (30)	6 (40)	0 (0)
Both, *n* (%)	2 (10)	1 (7)	1 (20)
Hemoglobin (g/dL) median (range)	12.3 (6.6–14.7)	12.2 (6.6–13.7)	13.7 (12.3–14.7)
Beta2 microglobulin (mg/mL) median (range)	2.8 (2.0–6.5)	3 (2.0–6.5)	2.3 (2.0–2.4)
Lactate dehydrogenase (U/L, UL^a^ 480) median (range)	165 (85–560)	165 (85–560)	179 (156–200)
Presence of BJ^b^ proteinuria, *n* (%)	8 (40)	7 (47)	1 (20)
Presence of immunoparesis, *n* (%)	4 (20)	4 (27)	0 (0)

^a^ Upper laboratory limit.

^b^ Bence Jones.

### 2.2 Next‐Generation Sequencing

NGS for DNA analysis was performed on 20 patients by conducting high‐throughput targeted AmpliSeq NGS. The resulting Ion Ampliseq Assay (Thermo Fisher Scientific) targeted 117 genes: 106 genes, including *MYD88* and *CXCR4* were targeted by the On‐Demand panel IAD205141, containing 3134 amplicons and run on a 530 ion chip (provides around 15–20 million reads) with a mean coverage of 7000X; 11 genes were targeted by the MTO panel IAD204582_182, (as listed in Table S1), a second smaller panel, containing 478 amplicons, run on a 520 ion chip (provides around 6–8 million reads) with a mean coverage of 19,500. Some of the 117 genes were chosen based on the differentially expressed genes highlighted in our previous study [16].

This assay provides accurate detection of DNA variants using as little as 10 ng of input nucleic acid extracted from BM CD19+ or CD138+ cells. Library preparation was performed automated by Ion Torrent Chef System (Thermo Fisher Scientific) using the Ion Ampliseq Custom or Made‐to‐order 2x Primer Pool 1 and 2 (Thermo Fisher Scientific) according to the Ion AmpliSeq Kit for Chef DL8 protocol, and templating was performed manually on the Ion Chef System kit (Thermo Fisher Scientific, #A34461). DNA libraries have been diluted to a concentration of approximately 40 pM and then run on the chip 530 (Thermo Fisher Scientific, #A27763) or two libraries were pooled together in the same ratio and run on the chip 520 (Thermo Fisher Scientific, #A27761). Sequencing was performed on the Ion S5 System Instrument (Ion Torrent, Thermo Fisher Scientific). Sequencing results were preliminary analyzed using Ion Torrent Suite v 5.12.1. Data analysis was performed using integrated Ion Reporter Software for variant calling, and Ion Torrent Oncomine Reporter. The resulting reports included detailed information about clinical trials, guidelines, and drug labels, as well as narratives describing the clinical context for the genes with detected variants. We examined MAFs for gene variants filtered by the software for each patient, comparing the frequencies of mutated genes in the BM CD19+ and CD138+ cells from WM/sWM and IgM MGUS patients.

## 3 Results

**FIGURE 1:**
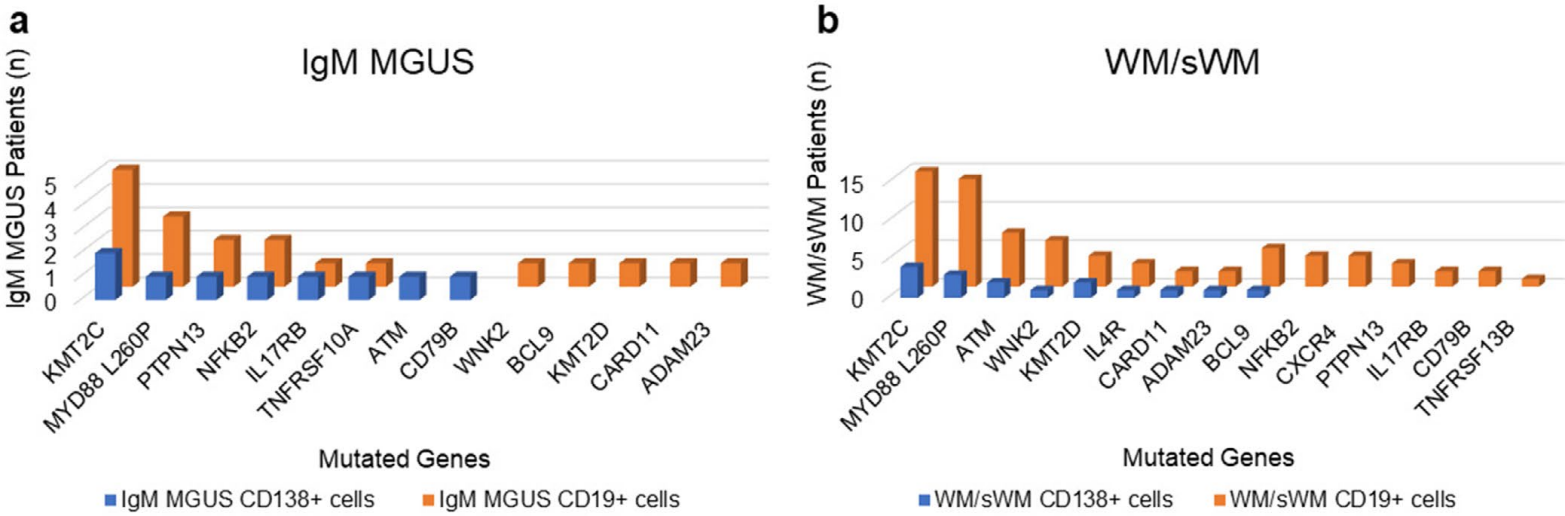
Mutational landscape of IgM MGUS (a) and WM/sWM (b) patients obtained by high‐throughput targeted sequencing. Graphical representation of the frequency of recurrently mutated genes obtained from NGS analysis, according to the cell fraction analyzed, CD19+ (orange) and CD138+ (blue) cells.

The mutational landscape of IgM MGUS and WM/sWM patients resulting from high‐throughput targeted sequencing on CD19+ and CD138+ cells, highlighted genetic aberrations in multiple genes, including the previously described recurrent mutations in *MYD88* (L260P alias L265P) found in 93% (14/15) of WM/sWM patients and in 60% (3/5) of IgM MGUS subjects. The *CXCR4* mutation (S338Ter) has been identified in 27% (4/15) of WM/sWM patients, exclusively within CD19+ cells, whereas it was absent in CD138+ cells of WM/sWM patients and in all IgM MGUS subjects. The mutational landscape of WM/sWM and IgM MGUS is summarized in Figure 1a,b.

**FIGURE 2:**
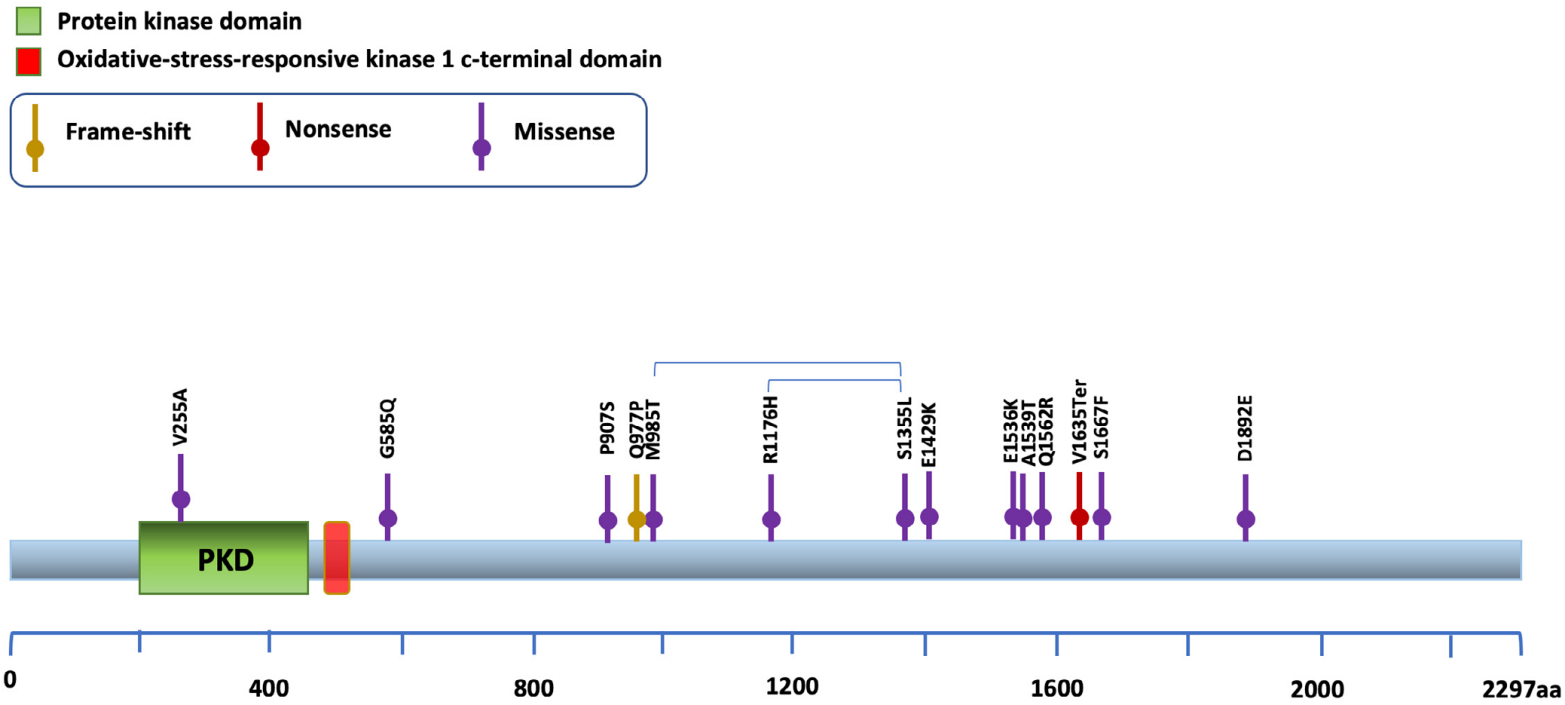
*WNK2* alteration in WM/sWM and IgM MGUS. Schematic shows the distribution of the identified *WNK2* somatic mutations.

Interestingly, we also identified new mutated genes, including *WNK2* (WNK Lysine‐Deficient Protein Kinase 2) somatic mutations affecting 47% of WM/sWM patients (7/15), for which a recurrent allelic variant (V1635Ter) was observed (Figure 2).

Moreover, one IgM MGUS (MGUS_25) patient showed a somatic hypermutated phenotype for *WNK2* in CD19+ cells but not in CD138+ cells (Table S2).

Furthermore, *BCL9* (BCL9 Transcription Coactivator) mutations were identified in 33% of WM/sWM patients (5/15), with a recurrent frameshift variant (P516Lfs) identified in 27% of WM/sWM patients (4/15), exclusively in the CD19+ cell fraction. The *BCL9* (P516Lfs) variant was also detected in the B cells of one IgM MGUS patient.

To be noted, *KMT2C* (also known as *MLL3*) exhibited a somatic hypermutated phenotype in both CD19+ and CD138+ cells with the *KMT2C* (I823T) allelic variant observed in all WM/sWM (15/15) and also in all IgM MGUS samples (5/5). The *KMT2C* mutations were not mutually exclusive with those involving *KMT2D* (mutated in 33% of WM/sWM patient and in one IgM MGUS patient) in our cohort of patients (Table S2). Moreover, sequencing evaluation revealed recurrent frameshift or missense mutations as follows: the *NFKB2* mutation (L473Afs) was found in three of 15 WM/sWM patients, exclusively within the CD19+ cell fraction, and in three of five IgM MGUS subjects, within CD19+ cells (2 of 3) and CD138+ cells (1 of 3). The *PTPN13* mutation (P1546Tfs) was identified in one of 15 WM/sWM patients (CD19+ cells) and in one of five IgM MGUS subjects (CD19+ cells). The *CARD11* mutation (S622del) was present in three of 15 WM/sWM patients, within CD19+ cells (2 of 3) and CD138+ cells (1 of 3), and in one IgM MGUS subject, exclusively within CD19+ cells (Table S2).

**FIGURE 3:**
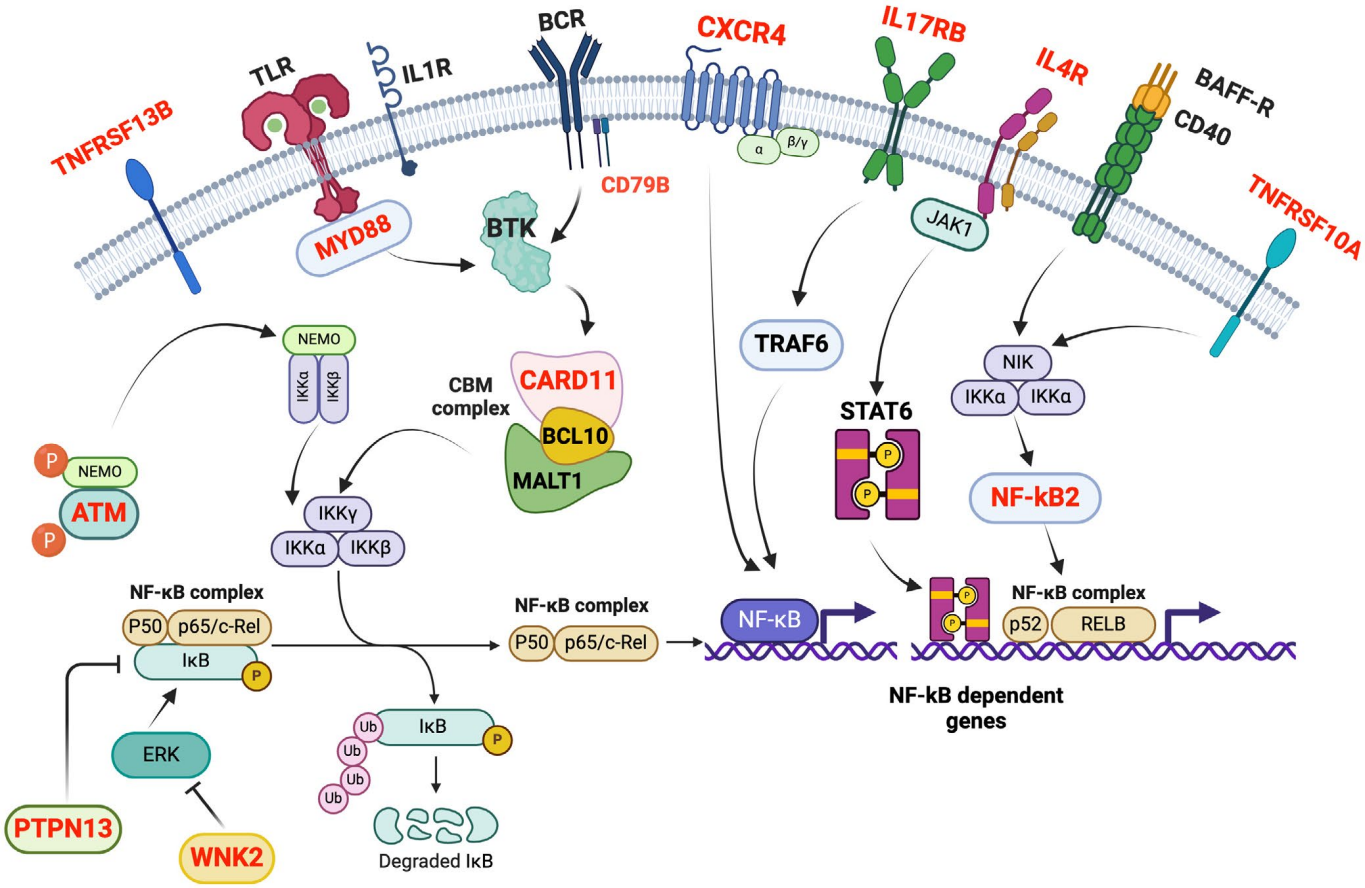
Activation and dysregulation of NF‐kB pathway in Waldenström Macroglobulinemia. In the schematic depicted, proteins encoded by corresponding genes identified as somatically mutated in this study are indicated in red, and it was found that they converge to activate the NF‐kB pathway, which is predominantly involved.

In addition, we observed that *ATM*, *NFKB2*, and *CARD11* belong to the NF‐kB pathway (Kegg hsa 04064), along with *MYD88*. Furthermore, *CXCR4*, *IL17RB*, *IL4R*, *TNFRSF10A*, and *TNFRSF13B* are part of the Cytokine‐Cytokine receptor Interactions Pathway (Kegg hsa 04060) (Figure 3). We listed all the mutated genes and the associated pathways in Table S3.

## 4 Discussion

The genetic framework outlined in this study offers a developing comprehension of the molecular mechanisms underlying the pathogenesis of WM. We investigated the genomic landscape by sequencing selected genes based on their known or possible role in WM. For this reason, we chose 117 genes from those which displayed known mutations in patients with WM and those with IgM MGUS, or those genes which were observed to be either upregulated or downregulated when comparing WM, IgM MGUS, and CTRLs [16].

In contrast to earlier genetic inquiries that documented singular genetic anomalies in WM, our research distinguished various recurring genetic abnormalities in WM and IgM MGUS.

The main goal was to ascertain the existence of genetic subtypes that include known mutations and uncover new genetic variants in both WM and IgM MGUS, hinting at a potential role in disease progression.

We previously investigated this cohort of patients through a transcriptome gene expression comparison among B cells from WM patients, IgM MGUS subjects, and healthy donors, which revealed several significantly differentially expressed genes (DEGs) (*n* = 2038). On the other hand, only 29 genes were identified as differently expressed by the gene expression profiling analysis among the CD138+ cells from WM patients, IgM MGUS subjects, and CTRLs. These genes encoded molecules involved in adherent and gap junction, cell adhesion, p53 signaling pathway, and calcium signaling pathway. Interestingly, we identified nine genes that displayed no significant differences in the expression levels between WM and IgM MGUS B cells compared to CTRLs [16].

In accordance with literature, we found that *MYD88* L265P was present in 93% of patients with sWM/WM and in 60% of patients with IgM MGUS. The *CXCR4* S338Ter mutation was detected exclusively in the CD19+ cells of 27% of sWM/WM patients, while it was absent in IgM MGUS patients.

Among the 117 sequenced genes, NGS analyses revealed new somatic mutations in genes that displayed mutations in previous studies: *ATM*, *CARD11*, *CD79B*, *KMT2C*, *KMT2D*, *NFKB2*, *PTPN13*, and *WNK2* in patients with sWM/WM as well as in IgM MGUS cases [2, 17, 18, 19, 20, 21, 22, 23] (Table S2). *ATM* frameshift and missense mutations were detected in sWM/WM patients and in one patient with IgM MGUS. The somatic S622del mutation in *CARD11* was found in sWM/WM patients and in one IgM MGUS patient.

We identified two previously described mutations, *CD79B* Y197N and *CD79B* Y197C, in one patient with sWM, and a new missense mutation, *CD79* Y197S in a patient with IgM MGUS [1, 11, 12, 24, 25].

Interestingly, all sWM/WM and IgM MGUS patients displayed a hypermutated *KMT2C* profile in both CD19+ and CD138+ cells as shown in Table S2. Notably, the missense *KMT2C* Y987H was detected in the CD138+ cells of sWM/WM and IgM MGUS patients, and in the CD19+ cells of sWM/WM patients, while the frameshift mutation *KMT2C* Y987Ter was observed in CD19+ cells of one WM patient. As described in previous studies, missense and frameshift *KMT2D* mutations were highly recurrent in WM patients [2, 11, 26]. Our study revealed new *KMT2D* missense and frameshift mutations in patients with sWM/WM and in one patient with IgM MGUS. Interestingly, *KMT2D* and *KMT2C* mutations dysregulate H3K4 methylation, resulting in NF‐κB activation [27].

NF‐kB is a multiprotein complex functioning as a transcription factor. The NF‐kB signaling pathway regulates the survival of normal and malignant B cells by controlling the expression of cell death regulatory genes. In this pathological context of WM, various mutations and extracellular stimuli activate the NF‐kB complex, influencing the transcription of target genes associated with cell survival and proliferation. The complex interaction of different molecular pathways contributes to the pathogenesis of Waldenström Macroglobulinemia, promoting the proliferation of B cells. Within NF‐kB signaling pathway, we identified a recurrent frameshift mutation *NFKB2* (L473AfsTer32) in sWM/WM patients and in IgM MGUS patients both with and without *MYD88* mutations.

In addition, we detected few *PTPN13* missense mutations and a recurrent frameshift mutation P1546Tfs in CD19+ cells of one IgM MGUS patient and in one sWM/WM patient, respectively.

Our study focused on *WNK2*, previously described by Guerrera et al., as it was identified as a tumor suppressor gene regulating ERK1/2, a prosurvival pathway active in WM. The authors demonstrated transcriptional and protein upregulation related to *MYD88* and *CXCR4* mutations. Specifically, the overexpression of spliced *WNK2* might contribute to *MYD88mut* WM oncogenesis [28]. Our previous gene expression profiling studies revealed *WNK2* upregulation in B cells of WM compared to IgM MGUS and CTRLs [16]. In our mutational analysis, we identified new missense and frameshift mutations of *WNK2* in both B cells and plasma cells of sWM/WM patients (Table S2). Interestingly, one patient with IgM MGUS exhibited a somatic hypermutated phenotype for *WNK2* in CD19+ cells (Table S2).

Additionally, we identified new somatic mutations in genes not previously described in WM and IgM MGUS namely *IL17RB*, *IL4R*, *ADAM23*, *BCL9*, *TNFRSF10A* (TNF Receptor Superfamily Member 10a), and *TNFRSF13B* (TNF Receptor Superfamily Member 13b).

Some authors demonstrated that IL17 induces significant WM cell proliferation [29]. We previously reported that *IL17RB* was upregulated in WM compared to IgM MGUS and CTRLs with high Fold Change (FC) [16]. Our mutational analysis revealed a new somatic mutation (L6V) in *IL17RB* within the CD19+ and CD138+ cells of one patient with IgM MGUS. Additionally, F278L and Q484Ter *IL17RB* mutations were identified in one sWM patient and in a WM patient, respectively.

As previously described, we confirmed that *IL4R* was downregulated in WM compared to IgM MGUS and CTRLs with high FC, but similar expression changes occurred between CTRLs and IgM MGUS [14, 16]. We detected two *IL4R* genomic variants in patients with sWM/WM.

Furthermore, NGS results revealed new *ADAM23* (R9G) missense mutations in one IgM MGUS patient and in two WM/sWM patients. To the best of our knowledge, the role of *ADAM23* in WM has not yet been elucidated, but the inactivation of this gene is associated with tumorigenesis in human cancers [30]. We previously observed the downregulation of *ADAM23* in WM and IgM MGUS compared to CTRLs, but no significant differences in the expression levels were observed between WM and IgM MGUS [16].

As far as we know, *BCL9* has not been previously associated with WM, but our analysis uncovered missense and frameshift mutations in both CD19+ and CD138+ cells of patients with sWM/WM and one IgM MGUS patient.

In this study, we also identified two new somatic mutations in *TNFRSF10A* and *TNFRSF13B*, both of which are involved in the cytokine–cytokine receptor interaction pathway, apoptosis, and p53 signaling pathway. In *TNFRSF10A*, the missense mutation G290R was found in B cells and plasma cells of one IgM MGUS patient, and the *TNFRSF13B* frameshift mutation V284A was present in B cells of one sWM patient.

Taken together, our findings highlight distinct patterns of mutations affecting new genes belonging to active pathways in WM, such as NF‐kappa B signaling pathway, JAK/STAT signaling pathway, cytokine–cytokine receptor interaction, P53 signaling pathway, and apoptosis.

However, it is important to acknowledge the limitations of this study, particularly the small number of patients with sWM/WM and those with IgM MGUS, which represents a subgroup of patients previously investigated for transcriptome profiling. On the other hand, we conducted NGS analysis, even in a limited number of patients, within B cells and plasma cells obtained from well‐diagnosed patients with WM, sWM, and IgM MGUS.

As expected, CD138+ cells showed a lower number of variants and a reduced percentage of mutations. A larger NGS‐based study of additional patients would be valuable to confirm these findings.

In conclusion, our study highlighted new mutated genes that could represent early or late event contributing to the transformation of IgM MGUS to WM. Further investigations into the significance of these newly identified somatic mutations in disease pathogenesis are merited, particularly in a broader cohort encompassing both patients diagnosed with WM and those with IgM MGUS.

## Author Contributions

**Alessandra Trojani:** conceptualization (lead), data curation (equal), formal analysis (supporting), funding acquisition (supporting), investigation (equal), methodology (equal), resources (supporting), software (supporting), supervision (supporting), validation (equal), visualization (equal), writing – original draft (lead), writing – review and editing (equal). **Alessandro Beghini:** conceptualization (equal), data curation (lead), formal analysis (lead), funding acquisition (supporting), investigation (equal), methodology (equal), project administration (supporting), resources (supporting), software (lead), supervision (supporting), validation (equal), visualization (equal), writing – original draft (equal), writing – review and editing (equal). **Luca Emanuele Bossi:** conceptualization (supporting), data curation (supporting), formal analysis (supporting), funding acquisition (supporting), investigation (equal), methodology (supporting), project administration (supporting), resources (supporting), software (supporting), supervision (supporting), validation (supporting), visualization (supporting), writing – original draft (supporting), writing – review and editing (supporting). **Marta Rachele Stefanucci:** conceptualization (supporting), data curation (supporting), formal analysis (supporting), funding acquisition (supporting), investigation (equal), methodology (supporting), project administration (supporting), resources (supporting), software (supporting), supervision (supporting), validation (supporting), visualization (supporting), writing – original draft (supporting), writing – review and editing (supporting). **Cassandra Palumbo:** conceptualization (supporting), data curation (supporting), formal analysis (supporting), funding acquisition (supporting), investigation (equal), methodology (supporting), project administration (supporting), software (supporting), supervision (supporting), validation (supporting), visualization (supporting), writing – original draft (supporting), writing – review and editing (supporting). **Antonino Greco:** conceptualization (supporting), data curation (supporting), formal analysis (supporting), funding acquisition (supporting), investigation (supporting), methodology (supporting), project administration (supporting), resources (equal), software (supporting), supervision (supporting), validation (supporting), visualization (supporting), writing – original draft (supporting), writing – review and editing (supporting). **Annamaria Frustaci:** conceptualization (supporting), data curation (supporting), formal analysis (supporting), funding acquisition (supporting), investigation (supporting), methodology (supporting), project administration (supporting), resources (equal), software (supporting), supervision (supporting), validation (supporting), visualization (supporting), writing – original draft (supporting), writing – review and editing (supporting). **Barbara Di Camillo:** conceptualization (supporting), data curation (equal), formal analysis (equal), funding acquisition (supporting), investigation (supporting), methodology (supporting), project administration (supporting), resources (supporting), software (equal), supervision (supporting), validation (supporting), visualization (supporting), writing – original draft (supporting), writing – review and editing (supporting). **Roberto Cairoli:** conceptualization (equal), data curation (equal), formal analysis (equal), funding acquisition (lead), investigation (equal), methodology (equal), project administration (lead), resources (lead), software (supporting), supervision (lead), validation (equal), visualization (equal), writing – original draft (equal), writing – review and editing (equal).

## Ethics Statement

The study was approved by the Ethics Committee ASST Grande Ospedale Metropolitano Niguarda (Milan, Italy) with the number 195‐2010‐009 on February 16, 2010, according to the Declaration of Helsinki.

## Consent

Informed consent was obtained from all subjects involved in the study.

## Conflicts of Interest

The authors declare no conflicts of interest.

## Supporting information


Table S1.

Table S2.

Table S3.

## Data Availability

This article includes the findings and interpretation of the results. Please contact the corresponding authors for additional data.

## References

[1] S. P. Treon , L. Xu , M. L. Guerrera , et al., “Genomic Landscape of Waldenström Macroglobulinemia and Its Impact on Treatment Strategies,” Journal of Clinical Oncology 38 (2020): 1198–1208. 32083995 10.1200/JCO.19.02314 PMC7351339

[2] L. Xu , Z. R. Hunter , G. Yang , et al., “MYD88 L265P in Waldenström Macroglobulinemia, Immunoglobulin M Monoclonal Gammopathy, and Other B‐Cell Lymphoproliferative Disorders Using Conventional and Quantitative Allele‐Specific Polymerase Chain Reaction,” Blood 121 (2013): 2051–2058. 23321251 10.1182/blood-2012-09-454355 PMC3596964

[3] C. Jiménez , E. Sebastián , M. Chillón , et al., “MYD88 L265P Is a Marker Highly Characteristic of, but Not Restricted to, Waldenström's Macroglobulinemia,” Leukemia 27 (2013): 1722–1728. 23446312 10.1038/leu.2013.62

[4] J. J. Castillo , C. Buske , J. Trotman , S. Sarosiek , and S. P. Treon , “Bruton Tyrosine Kinase Inhibitors in the Management of Waldenström Macroglobulinemia,” American Journal of Hematology 98 (2023): 338–347. 36415104 10.1002/ajh.26788 PMC10107762

[5] J. Khwaja , S. D'Sa , M. C. Minnema , M. J. Kersten , A. Wechalekar , and J. M. Vos , “IgM Monoclonal Gammopathies of Clinicalsignificance: Diagnosis and Management,” Haematologica 107 (2022): 2037–2050. 35770530 10.3324/haematol.2022.280953 PMC9425303

[6] D. F. Moreno , M. López‐Guerra , S. Paz , et al., “Prognostic Impact of MYD88 and CXCR4 Mutations Assessed by Droplet Digital Polymerase Chain Reaction in IgM Monoclonal Gammopathy of Undetermined Significance and Smouldering Waldenström Macroglobulinaemia,” British Journal of Haematology 200 (2023): 187–196. 36210485 10.1111/bjh.18502 PMC10092069

[7] S. Poulain , C. Roumier , A. Venet‐Caillault , et al., “Genomic Landscape of CXCR4 Mutations in Waldenström Macroglobulinemia,” Clinical Cancer Research 22 (2016): 1480–1488. 26490317 10.1158/1078-0432.CCR-15-0646

[8] J. Paludo and S. M. Ansell , “Advances in the Understanding of IgM Monoclonal Gammopathy of Undetermined Significance,” F1000Research 6 (2017): 2142. 29399323 10.12688/f1000research.12880.1 PMC5785715

[9] N. Sekiguchi , J. Nomoto , A. Nagata , et al., “Gene Expression Profile Signature of Aggressive Waldenström Macroglobulinemia With Chromosome 6q Deletion,” BioMed Research International 2018 (2018): 6728128. 30402490 10.1155/2018/6728128 PMC6193339

[10] L. Xu , N. Tsakmaklis , G. Yang , et al., “Acquired Mutations Associated With Ibrutinib Resistance in Waldenström Macroglobulinemia,” Blood 129 (2017): 2519–2525. 28235842 10.1182/blood-2017-01-761726 PMC7484977

[11] M. Varettoni , S. Zibellini , I. Defrancesco , et al., “Pattern of Somatic Mutations in Patients With Waldenström Macroglobulinemia or IgM Monoclonal Gammopathy of Undetermined Significance,” Haematologica 102 (2017): 2077–2085. 28983055 10.3324/haematol.2017.172718 PMC5709107

[12] C. Jiménez , S. Alonso‐Álvarez , M. Alcoceba , et al., “From Waldenström's Macroglobulinemia to Aggressive Diffuse Large B‐Cell Lymphoma: A Whole‐Exome Analysis of Abnormalities Leading to Transformation,” Blood Cancer Journal 7 (2017): e591. 28841204 10.1038/bcj.2017.72 PMC5596383

[13] Z. R. Hunter , L. Xu , G. Yang , et al., “Transcriptome Sequencing Reveals a Profile That Corresponds to Genomic Variants in Waldenström Macroglobulinemia,” Blood 128 (2016): 827–838. 27301862 10.1182/blood-2016-03-708263 PMC4982454

[14] N. C. Gutiérrez , E. M. Ocio , R. J. de Las , et al., “Gene Expression Profiling of B Lymphocytes and Plasma Cells From Waldenström's Macroglobulinemia: Comparison With Expression Patterns of the Same Cell Counterparts From Chronic Lymphocytic Leukemia, Multiple Myeloma and Normal Individuals,” Leukemia 21 (2007): 541–549. 17252022 10.1038/sj.leu.2404520

[15] B. T. Gaudette , B. Dwivedi , S. K. Chitta , et al., “Low Expression of Pro‐Apoptotic Bcl‐2 Family Proteins Sets the Apoptotic Threshold in Waldenström Macroglobulinemia,” Oncogene 35 (2016): 479–490. 25893290 10.1038/onc.2015.103 PMC4874246

[16] A. Trojani , B. Di Camillo , L. E. Bossi , et al., “Identification of a Candidate Gene Set Signature for the Risk of Progression in IgM MGUS to Smoldering/Symptomatic Waldenström Macroglobulinemia (WM) by a Comparative Transcriptome Analysis of B Cells and Plasma Cells,” Cancers (Basel) 13 (2021): 1837. 33921415 10.3390/cancers13081837 PMC8070603

[17] N. Lu , C. L. Neoh , Z. Ruan , et al., “Essential Thrombocythaemia With Concomitant Waldenström Macroglobulinaemia: Case Report and Literature Review,” Oncotargets and Therapy 13 (2020): 3431–3435. 32425546 10.2147/OTT.S245950 PMC7186880

[18] J. H. Lim , J. Q. Wang , F. Webb , et al., “Plasma Cells Arise From Differentiation of Clonal Lymphocytes and Secrete IgM in Waldenström Macroglobulinemia,” iScience 25 (2022): 104856. 35992066 10.1016/j.isci.2022.104856 PMC9389254

[19] C. I. E. Smith and J. A. Burger , “Resistance Mutations to BTK Inhibitors Originate From the NF‐κB but Not From the PI3K‐RAS‐MAPK Arm of the B Cell Receptor Signaling Pathway,” Frontiers in Immunology 12 (2021): 689472. 34177947 10.3389/fimmu.2021.689472 PMC8222783

[20] L. Ondrisova and M. Mraz , “Genetic and Non‐Genetic Mechanisms of Resistance to BCR Signaling Inhibitors in B Cell Malignancies,” Frontiers in Oncology 10 (2020): 591577. 33154951 10.3389/fonc.2020.591577 PMC7116322

[21] Z. R. Hunter , G. Yang , L. Xu , X. Liu , J. J. Castillo , and S. P. Treon , “Genomics, Signaling, and Treatment of Waldenström Macroglobulinemia,” Journal of Clinical Oncology 35 (2017): 994–1001. 28294689 10.1200/JCO.2016.71.0814

[22] J. Garcia‐Reyero , N. Martinez Magunacelaya , S. Gonzalez de Villambrosia , et al., “Diagnostic Value of Bone Marrow Core Biopsy Patterns in Lymphoplasmacytic Lymphoma/Waldenström Macroglobulinaemia and Description of Its Mutational Profiles by Targeted NGS,” Journal of Clinical Pathology 73 (2020): 571–577. 31980558 10.1136/jclinpath-2019-206282

[23] J. Kaur , S. S. Valisekka , M. Hameed , et al., “Monoclonal Gammopathy of Undetermined Significance: A Comprehensive Review,” Clinical Lymphoma, Myeloma & Leukemia 23 (2023): e195–e212. 10.1016/j.clml.2023.02.004 36966041

[24] X. R. Hunter , L. Xu , G. Yang , et al., “The Genomic Landscape of Waldenstrom Macroglobulinemia Is Characterized by Highly Recurring MYD88 and WHIM‐Like CXCR4 Mutations, and Small Somatic Deletions Associated With B‐Cell Lymphomagenesis,” Blood 123 (2014): 1637–1646. 24366360 10.1182/blood-2013-09-525808

[25] D. Drandi , P. Decruyenaere , M. Ferrante , F. Offner , J. Vandesompele , and S. Ferrero , “Nucleic Acid Biomarkers in Waldenström Macroglobulinemia and IgM‐MGUS: Current Insights and Clinical Relevance,” Diagnostics (Basel) 12 (2022): 969. 35454017 10.3390/diagnostics12040969 PMC9028641

[26] M. Varettoni , E. Boveri , S. Zibellini , et al., “Clinical and Molecular Characteristics of Lymphoplasmacytic Lymphoma Not Associated With an IgM Monoclonal Protein: A Multicentric Study of the Rete Ematologica Lombarda (REL) Network,” American Journal of Hematology 94 (2019): 1193–1199. 31378966 10.1002/ajh.25600

[27] T. Lu and G. R. Stark , “NF‐κB: Regulation by Methylation,” Cancer Research 75 (2015): 3692–3695. 26337909 10.1158/0008-5472.CAN-15-1022 PMC4573795

[28] M. L. Guerrera , Z. R. Hunter , K. Richardson , et al., “Aberrant Expression of Spliced WNK2 Is an Early Event in MYD88 Mutated WM That Activates ERK1/2 and Supports Tumor Growth,” 2023; Presented at: ASH2023 Congress; December 9–12, 2023; San Diego, CA, USA and Online. Poster P3303.

[29] R. H. Prabhala , D. Pelluru , M. Fulciniti , et al., “Interleukin‐17 and T_H_17 Pathway Supports Waldenstrom's Macroglobulinemia Cell‐Growth: Potential Therapeutic Implications,” Blood 116 (2010): 446.20460503

[30] J. C. Bolger and L. S. Young , “ADAM22 as a Prognostic and Therapeutic Drug Target in the Treatment of Endocrine‐Resistant Breast Cancer,” Vitamins and Hormones 93 (2013): 307–321. 23810013 10.1016/B978-0-12-416673-8.00014-9

