# Supplementary material for: Mutational Landscape of Bone Marrow CD19 and CD138 Cells in Waldenström Macroglobulinemia (WM) and IgM Monoclonal Gammopathy of Undetermined Significance (IgM MGUS)
Source: Cancer Med. 2024 Dec 23;13(24):e70525. doi: 10.1002/cam4.70525 (PMC11664121; doi:10.1002/cam4.70525)
Supplement: Supplementary file 1 — Table S1. [file CAM4-13-e70525-s001.docx]

| **Gene name** | |
| --- | --- |
| **ACTN1** | **IRF3** |
| **ADAM23** | **IRF4** |
| **ADARB1** | **ITGA2B** |
| **ADRB2** | **ITGAM** |
| **APBB2** | **ITGB3** |
| **ARID1A** | **ITPR1** |
| **ATP1B1** | **ITPR3** |
| **ATP2A2** | **JAK1** |
| **ATM** | **KITLG** |
| **BCL2** | **KDM6A** |
| **BCL9** | **KMT2D** |
| **BCL10** | **KMT2C** |
| **BCLAF1** | **MALT1** |
| **BTK** | **MAPK4** |
| **CACNA1D** | **MTOR** |
| **CARD11** | **MYB** |
| **CAV1** | **MYD88** |
| **CBLB** | **MYLK** |
| **CD226** | **NEGR1** |
| **CD4** | **NFKB2** |
| **CD44** | **NFKBIB** |
| **CD58** | **NRXN3** |
| **CD79A** | **OTUD4** |
| **CD79B** | **P2RY12** |
| **CD86** | **PF4** |
| **CDH2** | **PPIA** |
| **CDKN1B** | **PPP3CA** |
| **CFLAR** | **PRKCA** |
| **CNR1** | **PSEN1** |
| **CNTNAP2** | **PTGIR** |
| **CRKL** | **PTPN1** |
| **CUL9** | **PTPN13** |
| **CXCL12** | **PTPN4** |
| **CXCR4** | **PTPRJ** |
| **EML6** | **RAF1** |
| **EP300** | **RAG1** |
| **EZH2** | **RAPGEF1** |
| **FAF1** | **RYK** |
| **GP1BA** | **SERPINE1** |
| **GUCY2C** | **SLC8A1** |
| **HERC2** | **SMAD3** |
| **HIST1H1B** | **SMAD4** |
| **IGF1R** | **SOS1** |
| **IKZF2** | **STAT3** |
| **IL17RB** | **SYK** |
| **IL21R** | **SYTL3** |
| **IL2RA** | **TBL1XR1** |
| **IL4R** | **TFPI** |
| **IL6** | **TGFB2** |
| **THBS1** |  |
| **TLR8** |  |
| **TNFRSF10A** |  |
| **TNFRSF13B** |  |
| **TNIK** |  |
| **TP53BP1** |  |
| **TP53** |  |
| **TP63** |  |
| **TP73** |  |
| **TRAFD1** |  |
| **TRRAP** |  |
| **USP24** |  |
| **USP9X** |  |
| **VWF** |  |
| **WNK2** |  |
| **WNT3** |  |
| **XIAP** |  |
| **ZCCHC11** |  |
| **ZNF804A** |  |

**Supplementary Table 1**

List of 117 genes selected as targets for AmpliSeq next generation sequencing.
