# Supplementary material for: Mutational Landscape of Bone Marrow CD19 and CD138 Cells in Waldenström Macroglobulinemia (WM) and IgM Monoclonal Gammopathy of Undetermined Significance (IgM MGUS)
Source: Cancer Med. 2024 Dec 23;13(24):e70525. doi: 10.1002/cam4.70525 (PMC11664121; doi:10.1002/cam4.70525)
Supplement: Supplementary file 2 — Table S2. [file CAM4-13-e70525-s003.docx]

| ID patient | MYD88 | CXCR4 | WNK2 | BCL9 | NFKB2 | PTPN13 | CARD11 | KMT2C | CD79B | IL17RB | IL4R | ADAM23 | ATM | TNFRSF10A | TNFRSF13B | KMT2D |
| --- | --- | --- | --- | --- | --- | --- | --- | --- | --- | --- | --- | --- | --- | --- | --- | --- |
|  |  |  |  |  |  |  |  |  |  |  |  |  |  |  |  |  |
| MGUS_16 CD19 |  |  |  |  | **L473Afs 3.6%** |  |  | **G838S 48%**  **T316S 4.2%**  **D4240K 9.6%**  **P2412T 51%**  **I823T 2.8%** |  |  |  | **R9G 6.3%** |  |  |  | **D1825N 50%** |
| MGUS_25 CD19 | **L260P 36.6%** |  | **V255A 4.4%**  **P907S 5.8%**  **E1429K 5.1%**  **A1530T 4.1%**  **E1536K 12.2%**  **Q1562R 7.5%**  **S1667F 4.4%**  **D1892E 5.5%**  **c.3411 splice-1G>A** |  | **L473Afs 3.9%** |  |  | **G838S 52.9%**  **I823T 5.3%** |  | **L6V 49.8%** |  |  |  | **G290R 50.7%** |  |  |
| MGUS_25 CD138 | **L260P 4%** |  |  |  |  |  |  | **G838S 53.2%**  **I823T 3.2%** |  | **L6V 52.5%** |  |  |  | **G290R 51.6%** |  |  |
| MGUS_30 CD19 |  |  |  |  |  | **P1546Tfs 4.4%** | **S622del 4.6%** | **G838S 54.6%**  **I823T 4.7%** |  |  |  |  |  |  |  |  |
| MGUS_31 CD19 | **L260P 5.5%** |  |  |  |  | **T2386I 52%** |  | **G838S 41.9%**  **P2412T 49.5%**  **I823T 3%** |  |  |  |  |  |  |  |  |
| MGUS_31 CD138 |  |  |  |  | **L473Afs 5%** | **T2386I 50%** |  | **G838S 38.2%**  **P2412T 46%**  **I823T 2.9%**  **C988F 62.9%**  **Y987H 22.9%** | **Y197S 3.2%** |  |  |  | **S49C 46.8%** |  |  |  |
| MGUS_32 CD19 | **L260P 4.9%** |  |  | **P516Lfs 2.6%** |  |  |  | **G838S 61.3%**  **C3363F 50.6%**  **E765G 24.4%**  **Q755Ter 18%**  **S772L 70.4%**  **E616G 5.9%**  **I823T 4.9%**  **S3786Yfs 2.9%**  **T316S 7.6%**  **G315S 2.8%** |  |  |  |  |  |  |  |  |
| WM_02 CD19 | **L260P 46%** |  |  |  |  |  |  | **G838S 21%**  **C988F 71%**  **I823T 6.8%** |  |  |  |  |  |  |  |  |
| WM_04 CD19 | **L260P 46%** |  | **V1635Ter 27.7%** | **P516Lfs 3.9%** | **L473Afs 6.9%** | **P1546Tfs 12.6%** |  | **G838S 49.1%**  **C988F 40%**  **Y987Ter 5%**  **R973G 4.6%**  **T316S 10.3%**  **I823T 3.1%**  **Y987H 47.5%** |  |  |  |  |  |  |  | **P2557L 41.3%** |
| WM_15 CD19 | **L260P 55%** |  | **S1355L 43.6%**  **M985T 4.9%** |  | **P884Lfs 4.7%** |  | **S622del 4.8%** | **G838S 56.8%**  **T316S 8.2%**  **C988F 68.6%**  **I823T 5.3%**  **Y987H 22.9%**  **G315S 2.6%** |  | **F278L 53.8%** |  |  |  |  | **V284Afs 5.7%** |  |
| WM_35 CD19 | **L260P 49.1%** |  |  |  |  |  |  | **I823T 5.2%**  **G838S 27.9%**  **T316S 3%** |  |  |  |  |  |  |  |  |
| WM_37 CD19 | **L260P 44.7%** |  |  |  |  |  | **I1023M 46.7%** | **G838S 56.4%**  **I823T 3%** |  |  |  |  | **P1054R 51.8%** |  |  |  |
| WM_40 CD19 | **L260P 41.2%** | **S338Ter 42.2%** | **Q977Pfs 4.4%** |  |  |  |  | **G838S 54.4%**  **Q1787del 4.4%**  **I823T 3%** |  |  | **E376del 4.2%** |  |  |  |  |  |
| WM_41 CD19 | **L260P 30.8%** |  |  |  |  | **G1063E 50.1%** |  | **G838S 31.9%**  **I823T 5.2%**  **D1319H 50.5%**  **I993Nfs 14.3%**  **C988F 78.6%**  **Q668H 49%** |  | **Q484Ter 47.6%** |  |  |  |  |  |  |
| WM_45 CD19 | **L260P 49.4%** |  |  |  |  |  |  | **G838S 53.2%**  **I823T 5%**  **R2289C 53.6%**  **T316S 7.1%** |  |  | **P675S 41.3%** |  |  |  |  |  |
| WM_45 CD138 | **L260P**  **24.5%**  **E183Nfs 5.4%** |  |  |  |  |  |  | **G838S 51.5%**  **I823T 3.7%**  **R2289C 48.8%** |  |  | **P675S 45%** |  |  |  |  |  |
| WM_47 CD19 | **L260P**  **52.4%** | **S338Ter 46.5%** |  | **S19G 55.9%** |  |  |  | **G838S 25.2% I823T 9.2%** |  |  |  |  | **G2023R 52.5%** |  |  | **H832Q 61.3%** |
| WM_47 CD138 | **L260P**  **44.1%** | **S338Ter 46.6%** | **V1635Ter 35.2%** | **S19G 50.9%** |  |  |  | **G838S 22%**  **I823T 7.6%**  **T316S 5.6%** |  |  |  |  | **G2023R 52.1%** |  |  | **H832Q 64.7%** |
| WM_48 CD19 | **L260P**  **58.3%** |  | **R1176H 3%**  **S1355L 43.3%**  **M1708I 3.4%**  **P2202L 3.6%** | **P516Lfs 6.6%** | **L473Afs 4.1%** | **S1343Fs 4.6%**  **W2303Ter 3.3%** |  | **G838S 42.5%**  **I823T 8.5%**  **R2884Q 4.2%**  **R2296H 3.3%**  **P1316S 4.7%**  **Q642Ter 4.1%**  **G368E 4.4%**  **D1460N 4.1%**  **W1056Ter 6.5%**  **P40L 5.3%**  **Q13Ter 3.9%** | **Y197C 18.9%**  **Y197N 5.6%** |  |  |  | **W1750Ter 3.8%** |  |  |  |
| WM_49 CD19 | **L260P**  **45.5%** |  |  |  |  |  | **S622del 4.3%** | **G838S 37.2%**  **I823T 6.1%** |  |  |  |  | **L1420F 40.3%** |  |  | **A2214T 4%** |
| WM_50 CD19 | **L260P**  **19.4%** |  |  | **P516Lfs 10%** | **L473Afs 6.6%** |  |  | **G838S 39.6%**  **I823T 5.6%**  **T316S 6.1%** |  |  |  |  | **F858L 42.9%**  **P1054R 51.7%** |  |  |  |
| WM_50 CD138 | **L260P**  **16.3%** |  |  |  |  |  | **S622del 4.9%** | **G838S 41.4%**  **I823T 3.2%**  **T316S 4.4%**  **C988F 59.7%**  **Y987H 36.8%** |  |  |  |  | **F858L 48.3%**  **P1054R 51.4%** |  |  | **Q3905del 6.3%**  **L449M 4.6%** |
| WM_51 CD19 |  |  | **M985T 5.1%** |  |  |  |  | **G838S 42.9%**  **I823T 3.7%** |  |  |  | **S125A 49.1%** |  |  |  |  |
| WM_51 CD138 |  |  |  |  |  |  |  | **G838S 41.8%**  **I823T 2.6%**  **T316S 6.5%** |  |  |  | **S125A 48.8%** |  |  |  |  |
| WM_52 CD19 | **L260P**  **13.1%** | **S338Ter 14.8%** | **V1635Ter 34.5%** | **P516Lfs 2.2%** |  |  |  | **G838S 58.2%**  **T316S 6.6%** |  |  |  | **L117I 12.8%** | **R23Q 48.7%** |  |  | **R746P 28.6%**  **P692T 52.5%** |
| WM_53 CD19 | **L260P**  **23.6%** | **S338Ter 17.4%** |  |  |  |  |  | **G838S 43.1%**  **I823T 6.3%** |  |  | **P675S 42.2%** |  | **P604S 47.2%**  **S978P 56.2%**  **F1463C 51.4%** |  |  |  |

Supplementary Table 2. Mutations with respective Variant allele frequency (VAF) in BM CD19+ and CD138+ cells of WM/sWM and IgM MGUS patients.
