# Supplementary material for: Mutational Landscape of Bone Marrow CD19 and CD138 Cells in Waldenström Macroglobulinemia (WM) and IgM Monoclonal Gammopathy of Undetermined Significance (IgM MGUS)
Source: Cancer Med. 2024 Dec 23;13(24):e70525. doi: 10.1002/cam4.70525 (PMC11664121; doi:10.1002/cam4.70525)
Supplement: Supplementary file 3 — Table S3. [file CAM4-13-e70525-s002.docx]

| KEGG pathway/role | Gene symbol |
| --- | --- |
| NF-kB signalling (hsa 04064) | *CARD11*, *NFKB2*, *MYD88*, *ATM* |
| Cell cycle (hsa 04110) | *ATM* |
| P53 signalling pathway (has 04115) | *ATM*, *TNFRSF10A* |
| Apoptosis (hsa 04210) | *ATM*, *TNFRSF10A*, *PTPN13* |
| B cell receptor signalling pathway (hsa 04662) | *CD79B*, *CARD11* |
| Cytokine-Cytokine Receptor Interactions Pathway (hsa 04060) | *CXCR4*, *IL17RB*, *IL4R*, *TNFRSF10A*, *TNFRSF13B* |
| Lysine degradation (hsa 00310) | *KMT2C*, *KMT2D* |
| JAK-STAT signalling pathway (hsa 04360) | *IL4R* |
| Cell adhesion molecule | *ADAM23* |

**Supplementary table 3.** Mutated genes and their associated Kegg pathways.
